# Subacute Rumen Acidosis in Greek Dairy Sheep: Prevalence, Impact and Colorimetry Management

**DOI:** 10.3390/ani14142061

**Published:** 2024-07-14

**Authors:** Nikolaos Voulgarakis, Dimitrios A. Gougoulis, Dimitra Psalla, Georgios I. Papakonstantinou, Konstantinos Katsoulis, Mariana Angelidou-Tsifida, Labrini V. Athanasiou, Vasileios G. Papatsiros, Georgios Christodoulopoulos

**Affiliations:** 1 Clinical Veterinary Medicine Department, Faculty of Veterinary Medicine, University of Thessaly, 431 00 Karditsa, Greece; nvoulgarakis@vet.uth.gr (N.V.); dgoug@vet.uth.gr (D.A.G.); geopapak@vet.uth.gr (G.I.P.); , vpapatsiros@vet.uth.gr (V.G.P.); 2 Laboratory of Pathology, School of Veterinary Medicine, Aristotle University of Thessaloniki, 541 24 Thessaloniki, Greece; dpsalla@vet.auth.gr (D.P.); mtangelid@vet.auth.gr (M.A.-T.); 3 Department of Animal Husbandry and Nutrition, Faculty of Veterinary Medicine, School of Health Sciences, University of Thessaly, 431 00 Karditsa, Greece; kkatsoulis@vet.uth.gr; 4 Department of Animal Science, Agricultural University of Athens, 118 55 Athens, Greece

**Keywords:** subacute rumen acidosis, rumen mucosa colorimetry, dairy sheep, milk fat depression, epidemiology, Greece

## Abstract

**Simple Summary:**

Subacute rumen acidosis (SARA) is a significant clinical issue in high-yield livestock, including dairy sheep, and it is characterized by rumen pH dropping below 5.8 for at least four hours daily. This condition leads to economic losses due to reduced milk production and lower milk fat content. High-concentrate, low-fiber diets increase the risk of SARA by disrupting rumen fermentation. Diagnosing SARA is challenging, with rumen fluid pH measurement being the gold standard. This study investigated the prevalence of SARA and its impact in forty-two Greek dairy sheep flocks, as well as the potential of computerized rumen colorimetry as a diagnostic tool *post mortem*. Over nine months, rumen pH and milk composition were monitored, and rumen wall samples were analyzed for color and histological changes. The results showed increased epithelium thickness and discoloration in SARA-affected sheep. This study highlights the prevalence of SARA in the modern intensive dairy sheep industry, underscores the need for balanced feeding strategies, and suggests computerized rumen colorimetry as a promising tool for SARA detection and management in dairy sheep farms.

**Abstract:**

Subacute rumen acidosis (SARA) is a current issue in intensive livestock farming, and it is particularly associated with feeding high-concentrate diets. This study investigated the prevalence and impact of SARA in forty-two Greek dairy sheep flocks by recording rumen pH, milk composition, and milk yield over a period of nine months. Moreover, it explored the use of computerized rumen colorimetry as a management and diagnostic tool for SARA in dairy sheep. In culled ewes, computerized rumen mucosal colorimetry was applied, and rumen wall samples taken for histological examination. SARA cases were identified in 19 farms (45%, *n* = 42). Farms with SARA cases had lower milk fat levels, while milk yield and milk protein levels did not differ based on the SARA status of the farms. In culled ewes, rumen color was significantly associated with the flock’s SARA status, and affected ewes showed increased thickness in non-keratinized and total epithelial layers. It was concluded that computerized rumen mucosal colorimetry in aged, culled ewes shows promise as an indicator, *post mortem*, of SARA present in dairy sheep flocks whose impact can be minimized by making significant changes in dietary management.

## 1. Introduction

In high-yield livestock farming, rumen acidosis is a common clinical issue [1,2]. It occurs in two forms: acute, which is characterized by a rumen pH below 5.0, and subacute (subacute ruminal acidosis—SARA), which is characterized by rumen pH being lower than 5.8 for at least four hours per day [3,4,5]. In contrast to acute rumen acidosis, SARA does not cause acute rumenitis, and any potential mucosal degenerative changes either do not occur or are reversible. However, SARA still leads to economic losses due to reduced milk production and lower milk fat content [5,6].

Feeding high-concentrate diets with low percentages of dietary fiber for extended periods increases the risk of subacute rumen acidosis, negatively affecting both milk production and fat concentration [7,8,9]. The subsequent lower rumen pH disrupts fermentation and bacterial synthesis, leading to an increased production of long-chain fatty acids (LCFAs), which hinders milk fat synthesis in the udder [10,11].

Several hematobiochemical parameters have been suggested as diagnostic markers for SARA in small ruminants, but none have proven to be entirely accurate [5,12]. Rumen fluid pH measurement is the gold standard, with a pH between 5.2 and 5.8 for more than four hours per day indicating SARA in intensive small ruminant livestock farming [5,12].

Rumen acidosis, marked by rumen wall discoloration, especially in subacute rumenitis, manifests as a gray to dark discoloration [13,14,15,16]. In cattle, the objective estimation of color using spectrophotometers or colorimeters through Red, Green, and Blue (RGB) analysis has emerged as a potential method for assessing various health issues [17,18,19].

Sheep farming in Greece is a significant component of the country’s agricultural landscape, with approximately 7.3 million animals across 83,000 holdings [20,21]. Sheep farming in Greece is distinguished by its high dairy specialization, with 93% of sheep being dedicated to milk production. The primary use of this milk is for the production of feta cheese, a Protected Designation of Origin product and the most important export of primary Greek agriculture [22]. Despite its importance, the Greek sheep population has seen a decline of over 10% since 2010. This reduction has been marked by a decrease in the number of small and medium-sized holdings, which have been increasingly replaced by fewer, larger operations housing more than 500 ewes each. These larger enterprises predominantly employ an intensive indoor production system, relying heavily on imported concentrate feeds, supplemented by locally produced hay and sometimes ensilage. The current sheep population mainly consists of crossbreeds between various indigenous sheep and foreign breeds, such as mainly Lacaune and secondary Friesian, of which sheep of Lacaune cross adapt especially well to the intensive farming methods now prevalent in Greece.

While SARA has been extensively studied in other livestock, research on this topic in dairy sheep is limited [23]. This study aims to address this gap by evaluating the prevalence and impact of SARA in Greek dairy sheep farming and by assessing the effectiveness of computerized rumen colorimetry—a tool developed by the authors in a previous publication [19]—for managing and detecting SARA in these farms.

## 2. Materials and Methods

### 2.1. Selection of Farms

Forty-two dairy sheep flocks, each consisting of 500–1000 milking ewes, were carefully selected to participate in this study. These farms are situated in Thessaly, a central region of Greece. Inclusion criteria required farms to be within reach of the Department of Clinical Veterinary Medicine, Faculty of Veterinary Science, University of Thessaly, Greece. Farms also needed to have crossbreed ewes of Lacaune with the local Karagouniko breed, maintain a stable ration composition throughout the entire milking period, and be confirmed as free of paratuberculosis and ovine progressive pneumonia, as evidenced by the department’s archives. The ration composition on the 42 farms consisted of a specified amount of complementary concentrate mixture given per head and cereal hay provided *ad libitum*. Throughout the 9-month trial period, the complementary feed remained unchanged.

### 2.2. Experimental Design

The selected farms were monitored every two months for rumen pH throughout the entire milking period, spanning approximately nine months. Five visits were conducted at each farm during the first 10 days of the 1st, 3rd, 5th, 7th, and 9th month post-partum.

At each visit, six ewes were randomly chosen and assessed for rumen pH, ensuring they had not been previously selected for rumen pH measurement to prioritize animal welfare. Rumen fluid sampling took place four hours after the morning administration of concentrated feed. Simultaneously, milk samples were gathered from the bulk milk tank during morning visits. Additionally, data on daily milk yield were sourced from the farmers’ records.

Furthermore, between July and August 2021, during the culling of aged ewes from the above 42 farms, rumen sampling was performed in slaughterhouses. Due to economic constraints, a total of 280 ewes were included in the sampling, with at least 6 ewes from each farm.

### 2.3. Sample Size Justification

According to the existing literature, the prevalence of subacute rumen acidosis (SARA) in dairy sheep is approximately 12% [24]. To ensure a desired level of accuracy with a margin of error (e) of ±0.05 and a 95% confidence interval (Z = 1.96), the estimated sample size (n) was calculated using the formula *n* = (Z^2^ × P × (1 − P))/e², yielding a value of 162. This study exceeded this calculated sample size requirement during both farm and slaughterhouse sampling phases.

### 2.4. Ethical Approval

This study adhered to established animal welfare regulations (Directive 98/58/EC) and obtained approval from the Ethics Committee of the Faculty of Veterinary Science at the University of Thessaly, Greece (approval number: 152/16 May 2021). No modifications were made to the animals’ diets or treatments for experimental purposes during the entire duration of this study.

### 2.5. Clinical and Laboratory Procedures

#### 2.5.1. Rumenocentesis and Rumen pH Measurement

Rumenocentesis, conducted without sedation, was employed for the collection of rumen fluid samples, following established methodologies from prior studies [25]. The procedure included an external abdominal puncture using a 16 G (105 mm) needle (Intralune PP, Vygon, Écouen, France) in the left abdominal region, specifically targeting the ventral sac of the rumen and aspirating the fluid with a 50 mL syringe [5,26]. The point for rumenocentesis was identified at the intersection of two lines: one horizontal, passing through the stifle joint, and one vertical, passing through the midpoint between the base of the last rib and the hip bone. The obtained rumen fluid underwent immediate pH determination, utilizing a digital portable pH meter (HI-2002 Edge pH Meter, Hanna instruments, Leighton Buzzard, UK).

#### 2.5.2. Milk Analyses

To maintain sample quality, milk samples were refrigerated at 4 °C during transportation to the laboratory. On the same day of collection, the milk composition, including fat, protein, lactose, and total solids’ content, was analyzed using an infrared analysis technique with the assistance of an FTIR interferometer (Milkoscan FT6000 Analyzer, Foss Electric, Hilleroed, Denmark). The annual average values for the mentioned milk components were calculated based on the five measurements in each farm, along with the monthly milk yield, following basic arithmetic procedures [27].

#### 2.5.3. Rumen Wall Sampling and Image Acquisition

The sampling procedure for the rumen wall followed a standardized protocol, as detailed by Voulgarakis et al. [19], ensuring that the same anatomical area of the rumen wall was sampled each time. This standardized protocol, developed specifically for this purpose, was based on certain measurements and was created by the Clinical Veterinary Medicine Department, Faculty of Veterinary Science, University of Thessaly. Finally, a 5 cm × 5 cm square section of the rumen wall was excised for sampling. After excision, the sample underwent thorough washing with water to eliminate any visible remnants of rumen content. For image acquisition, the collected samples were placed on a specific desk 35–37 mm between the lens and ruminal epithelium surface, and digital gross images were taken. A digital camera (NIKON COOLPIX L330, Tokio, Japan) was used with an EF-S 60 mm macro lens (NIKKOR Wide-Angle Optical Zoom Lens 21×, Nikon Corporation, Tokio, Japan) with standard lighting conditions [halogen lighting (4 matt bulbs, 18 W each, color temperature of 2800 K, color rendering index of 90 to 100)] and settings (manual operation mode with an f/3.5 aperture, 1/30 s shutter speed, and ISO range on 160). The images were saved in raw format [19]. The sample was then placed in a container with a 10% formalin solution for subsequent histological examination.

#### 2.5.4. Rumen Wall Image Color Analysis 

The quantification of rumen mucosa color was determined using software applications, specifically RGB Color Detector 3.0.7 [28] and CIELab 1.0 [29].

In the RGB application, the analyzed color components included Red, Green, and Blue (RGB), Value (V), Lightness (L), Hue, Saturation, and Value (HSV), and Hue, Saturation, and Lightness (HSL). Additionally, the summary of the Red, Green, and Blue color components was computed and labeled as RGB-SUM [17]. Using the CIELab software 1.0, the L (color lightness), a (redness/greenness of the color), and b (yellowness/blueness of the color) color components were assessed [29].

For processing and analyzing the image, a 1 × 1 cm area within the region of interest was randomly selected from each sample. For each rumen wall sample, average values of these color components were calculated from nine distinct spot measurements, and the resulting average value was employed for subsequent statistical analysis. It is essential to note that, although the L, a, and b values are technically denoted as L*, a*, and b*, respectively, for simplicity, this text omits the asterisks.

#### 2.5.5. Histological Examination 

**Figure 1:**
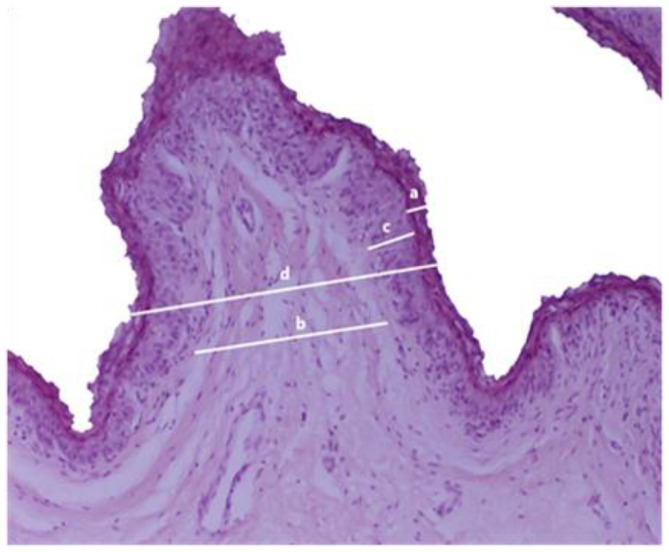
Measurements of the ewe rumen papillae: (a) Stratum corneum thickness. (b) Width of connective tissue. (c) Thickness of non-keratinized epithelium. (d) Total epithelium thickness. (Hematoxylin-eosin staining; magnification ×10).

For the histological examinations, the rumen wall samples were cut into 1 cm × 1 cm size particles. Tissues were fixed in 10% formalin buffer for 48 to 72 h. The processed tissues were dehydrated by being immersed in a series of alcohol solutions and embedded in paraffin sections (Paraplast Plus^®^, Kendall, UK). Afterwards, 4 μm thick sections were obtained via semi-automatic precision microtome (CUT 5062, SLEE medical GmbH, Nieder-Olm, Germany), dewaxed and stained with hematoxylin and eosin (H&E), for histopathological evaluation. From each sample, randomly selected fields with a ×10 objective were captured using a microscope (Nikon eclipse 50i, Tokyo, Japan)-coupled camera (Nikon, Digital sight DS-L1, Tokyo, Japan). ImageJ software 1.53 was utilized to measure the thickness of the stratum corneum, connective tissue, non-keratinized epithelium, and total epithelium in each photomicrograph (Figure 1).

### 2.6. Statistical Analysis

The consistency of rumen pH measurements over time in each farm was examined using the intraclass correlation coefficient (ICC). Continuous variables in the dataset, including pH values, milk parameters (such as yield, fat, protein, lactose, and total solids’ content), color measurements, and characteristics of the rumen epithelium, were checked for normal distribution. When necessary, log transformations were applied to address any observed skewness in the data. To explore potential differences in these variables among farms with low, moderate, and high SARA prevalence, a one-way analysis of variance (ANOVA) was conducted using IBM SPSS Statistics version 29.0.0.0 (241). The results were presented using mean and standard deviation (SD) as measures of central tendency and variability, respectively. The analysis was performed using IBM SPSS Statistics version 29.0.0.0 (241), with a significance level of 0.05 used to determine statistical significance.

## 3. Results

All animals involved in this study completed the entire monitoring period, without any adverse effects.

The 42 farms were categorized into three groups based on the number of rumen pH measurements below 5.8 out of 30 measurements conducted in each farm [*n* = 5 visits (×) 6 ewes each visit = 30 measurements per farm]. The categories and the number of farms in each category were as follows:

- Category 1: Farms with a low prevalence of SARA, including 17 farms (41% of the total), with each farm exhibiting only 0–2 cases of pH < 5.8 in the 30 measurements.
- Category 2: Farms with a moderate prevalence of SARA, comprising 19 farms (45% of the total), with each farm showing 3–9 cases of pH < 5.8 in the 30 measurements.
- Category 3: Farms with a high prevalence of SARA, consisting of 6 farms (14% of the total), with each farm having 10–14 cases of pH < 5.8 in the 30 measurements.

Table 1 shows the chemical composition and feeding amount per head of concentrate mixtures used on farms. Although there are arithmetic differences between the three farm categories, these differences were not statistically significant. 

Throughout the farming sampling period, the mean pH values of the participating farms, with six randomly selected ewes each time, ranged from 5.73 to 5.93, yielding a mean pH of 5.83 (standard deviation (SD) = 0.06). Specifically, in Category 1 farms, the pH range was between 5.87 and 5.93, with a mean pH of 5.89 (SD = 0.01). In Category 2, the pH range was from 5.78 to 5.84, with a mean pH of 5.81 (SD = 0.02). Lastly, in Category 3, the pH range was recorded between 5.73 and 5.76, with a mean pH of 5.74 (SD = 0.01). The sample size for each category was consistent. Post hoc analysis using the Bonferroni correction revealed significant differences among all possible pairwise comparisons between the three farm categories (*p* < 0.001) (Table 2).

In the abattoir, post hoc analysis revealed significant differences in the color components among all possible pairwise comparisons of the three farm categories, with the exception of the a component across all farm categories and the b component between Category 1 and Category 2 (*p* < 0.001) (Table 3).

### 3.1. Milk Analysis

Milk fat content varied across farm categories, with Category 1 having the highest average fat content and Category 3 the lowest, as shown in Table 2. Post hoc analysis indicated significant differences between Category 1 and Category 2, as well as between Category 1 and Category 3 (*p* < 0.001). As a direct consequence of these fat differences, there were also variations in the milk fat/milk protein ratio, with Category 1 having the highest ratio and Category 3 the lowest. Milk yield and milk protein did not vary between farm categories; however, small but statistically significant differences were found in the concentration of lactose and the total solids’ content (Table 2).

### 3.2. Color Measurement

Pairwise comparisons of mean L component values among all categories (1, 2, 3) revealed statistically significant differences (*p* < 0.001). Specifically, Category 1 exhibited the highest mean L value, whereas Category 3 reported the lowest mean L value, as reported in Table 3. Significant differences were also observed in the mean values of the b component between Category 1 and Category 3, as well as between Category 2 and Category 3, as shown in Table 3. However, no statistically significant differences were detected in the mean values of the a component across all possible comparisons (Table 3).

Regarding the color measurement using the RBG analysis system, the mean values of Red, Green, Blue, and RGB-SUM were found to be significantly higher in Category 1 when compared to the mean values of Category 2 and Category 3 samples. Similarly, Category 2 exhibited significantly higher mean values compared to Category 3. Furthermore, significant differences were observed for the HSV value and HSL value among all possible comparisons, with Category 1 having the highest values compared to Category 2 and Category 3. Lastly, Category 2 revealed significantly higher mean values compared to Category 3 (Table 3).

### 3.3. Ruminal Epithelium Histopathology and Histomorphometry

Histological analyses revealed no abnormal findings in the rumen epithelium samples, and parakeratosis was not observed. The darkest coloration was confined solely to the keratinized layer in these samples.

According to the statistical analysis, Category 3 exhibited a significantly higher thickness in non-keratinized epithelium thickness and total epithelium thickness in contrast to Category 2 and Category 1, as shown in Table 4. Additionally, the non-keratinized epithelium thickness and total epithelium thickness were significantly higher in samples of Category 2 compared to samples of Category 1. However, no significant differences were observed in stratum corneum thickness and connective tissue width in any of the possible comparisons (Table 4).

## 4. Discussion

This research represents the first attempt to investigate the epidemiology of SARA in dairy sheep farms in Greece. The findings strongly indicate that SARA is a common disorder in the modern Greek dairy sheep industry. These results are unsurprising given the typical nutritional management practices on farms. Ewes were provided with a complementary concentrate mixture that exceeded 1500 g per day throughout the milking period (see Table 1) and were also offered cereal hay *ad libitum*. Typically, ewes consume the allotted amount of the complementary concentrate mixture promptly, but the consumption of cereal hay often depends on its quality, leading to variable intake and potentially failing to meet the animals’ daily fiber requirements. It is known that feeding more than 500 g of cereals daily increases the risk of ruminal acidosis in sheep; therefore, the recommendation is that if more than 500 g is fed to a ewe, it should be in the form of a total mixed ration (TMR) rather than as a complementary feed [30].

The complementary concentrate mixtures used on the farms did not show significant differences in their chemical composition or feeding amounts (see Table 1). This was expected as feeding practices and ration formulation in modern Greek sheep farming are quite homogeneous. The lack of differences in the concentrate mixtures among the investigated farms underscores the causative role of hay in the cases of SARA documented in this research.

In this study, farms with a higher number of SARA cases (Categories 2 and 3) exhibited lower milk fat content. Lower ruminal pH is attributed to alterations in rumen fermentation and changes in the composition of rumen bacteria and fermentation patterns, leading to an increased production of specific LCFAs. These LCFAs, once absorbed, can inhibit milk fat synthesis in the udder [10,11]. Multiple studies, including those by Tao et al. [31], Zhang et al. [32], Li et al. [10], and Kitkas et al. [11], have consistently observed lower milk fat percentages in high-concentrate groups compared to low-concentrate groups in both dairy cows and goats. To the best of the authors’ knowledge, this research is the first to document this effect in dairy ewes. 

This research proposes a computerized measurement of rumen epithelium color as a promising method for monitoring SARA status. This technique can be easily applied during the slaughter of aged, culled ewes, providing health advisors with reliable data on SARA status in the farm. Currently, rumen pH measurement is considered the gold standard for diagnosing SARA in ruminants [33,34]. However, pH measurement can be influenced by various factors, including the method of measurement, sampling location, and timing [35,36]. Beyond pH measurements, diagnostic indicators such as milk fat percentage and the milk fat/protein ratio have been proposed for detecting SARA in dairy cows. Rapid weekly decreases in milk fat content of 0.3–0.5% or a milk fat/protein ratio below 1 suggest potential SARA cases [37,38,39,40]. However, these indexes have not been established for the dairy sheep industry, partly due to the ongoing genetic improvements in dairy sheep breeds. Additionally, chronic diseases such as progressive pneumonia and Johne’s disease are associated with decreases in milk fat content [41,42].

The existing literature indicates that the prevalence of SARA in dairy sheep is approximately 12%, but this study was conducted in Brazil [24]. This study exceeded the calculated sample size requirement based on this figure to account for the lack of extensive data from Greece and other Mediterranean countries.

The mean color values, measured by RGB and CIELab systems, were significantly lower in Category 3 samples compared to Categories 1 and 2, indicating darker discoloration of the ruminal epithelium. Extensive research and reviews on this topic, both in dairy and fattening lambs, have shown that feeding highly fermentable feed, which leads to reduced ruminal pH and increased accumulation of volatile fatty acids and lactic acid, is associated with a gray to dark coloration [13,14,15,16,43,44]. Similar results were reported in feedlot cattle by Voulgarakis et al. [19]. Unfortunately, as far as is known, no research data from dairy ewes have been published yet. Other factors contributing to the darkening of the ruminal mucosa include pelleting, fiber particle size, dietary iron levels, and the buffering system [43,45]. However, the generally accepted conclusion among sheep practitioners is that rumen pH plays a decisive role in the darkening of the keratinized layer in the ruminal epithelium. 

Histological findings showed that the darkening of the rumen epithelium’s color was due to the effect of rumen pH on the keratinized layer rather than parakeratosis. This is a notable finding as previous research attributed rumen epithelium darkening to parakeratosis [37].

Furthermore, an increase in the thickness of ruminal epithelium layers, particularly non-keratinized and total epithelium, was observed in animals from farms with a high incidence of SARA. Steele et al. [46] observed increased total epithelial thickness and stratum corneum thickness in animals fed a high-grain diet. Bo Trabi et al. [47] found that animals fed a low-grain non-pelleted TMR concentrate had higher total epithelium and stratum corneum thickness values compared to those fed a high-grain non-pelleted TMR concentrate or high-grain TMR in a pelleted form. Wang et al. [48] reported increased total epithelium and stratum corneum thickness values in animals fed a ground corn high-grain diet compared to a whole corn high-grain diet. No significant difference in stratum corneum thickness was found among the categories, but Category 3 samples had a greater stratum corneum length compared to Category 1 samples. Garcia Diaz et al. [49] reported higher total epithelium and stratum corneum thickness values but lower connective tissue width and non-keratinized thickness in animals fed a grain-based diet compared to those on an additive-supplemented grain-based diet. Lower rumen pH may increase the keratinized epithelium’s rate of replacement, stimulating non-keratinized epithelium growth to produce keratinized layers [19].

## 5. Conclusions

In conclusion, this study sheds light on the prevalence and impact of subacute rumen acidosis (SARA) in Greek dairy sheep flocks. Our findings highlight that SARA significantly affects dairy sheep farms, manifesting in notable changes in the rumen epithelium, which can be detected through computerized rumen colorimetry. The observed discoloration is indicative of acidic effects on the upper layers of the epithelium, rather than the previously suggested presence of parakeratosis.

The use of computerized rumen colorimetry in culled ewes as a diagnostic tool shows promise for assessing SARA status in dairy sheep farms. This technology provides farmers and health advisors with reliable data on SARA prevalence, enabling informed decisions to optimize feeding strategies and enhance herd health.

Looking ahead, further research and the development of comprehensive feeding practices are crucial. Strategies such as integrating concentrate mixtures into total mixed rations (TMRs) could mitigate SARA risks while promoting optimal milk production and ensuring animal welfare in dairy sheep farms.

## Figures and Tables

**Table 1:** Chemical composition and feeding amount per head (mean ± SD) of the complementary concentrate mixture used on farms.

Chemical Composition	Category 1 ^i^ (*n* = 17)	Category 2 ^ii^ (*n* = 19)	Category 3 ^iii^ (*n* = 6)	*p*-Value
ME (Mcal/kg DM)	2.65 ± 0.07	2.66 ± 0.07	2.66 ± 0.08	0.96
CP (% of DM)	16.06 ± 0.97	16.05 ± 0.97	16.50 ± 1.38	0.72
NDF (% of DM)	14.47 ± 0.80	14.42 ± 0.77	14.33 ± 1.21	0.95
ADF (% of DM)	5.94 ± 0.83	6.11 ± 0.81	6.17 ± 0.75	0.77
Feeding Amount (g/day)	1,682 ± 107	1,705 ± 118	1,758 ± 150	0.43

^i^ Farms with a low prevalence of SARA; ^ii^ farms with a moderate prevalence of SARA; ^iii^ farms with a high prevalence of SARA; ME = metabolizable energy; CP = crude protein; NDF = neutral detergent fiber; ADF = acid detergent fiber; DM = dry matter.

**Table 2:** Comparison of rumen pH, milk yield, and milk components (mean ± SD).

Farm Sampling Period	Category 1 ^i^ (*n* = 17)	Category 2 ^ii^ (*n* = 19)	Category 3 ^iii^ (*n* = 6)	*p*-Value
Rumen pH	5.89 ± 0.01	5.81 ± 0.02	5.74 ± 0.01	<0.001
Milk yield (liters/ewe/milking period)	412.0 ± 1.1	414.5 ± 2.1	415.1 ± 0.6	0.143
Milk fat content (%)	5.92 ± 0.14	5.69 ± 0.11	5.65 ± 0.06	<0.001
Milk protein content (%)	5.72 ± 0.10	5.72 ± 0.12	5.83 ± 0.05	0.064
Milk lactose content (%)	4.71 ± 0.09	4.81 ± 0.14	4.95 ± 0.16	0.001
Milk total solids’ content (%)	10.87 ± 0.23	11.06 ± 0.31	11.73 ± 0.40	<0.001
Milk fat/milk protein ratio	1.04 ± 0.02	0.99 ± 0.02	0.96 ± 0.01	<0.001

Significant difference at *p* ≤ 0.001; ^i^ farms with a low prevalence of SARA; ^ii^ farms with a moderate prevalence of SARA; ^iii^ farms with a high prevalence of SARA.

**Table 3:** Comparison of color components (mean ± SD) in rumen mucosa samples.

Color Components	Category 1 ^i^ (*n* = 111)	Category 2 ^ii^ (*n* = 124)	Category 3 ^iii^ (*n* = 45)	*p*-Value
L	39.93 ± 5.24	30.59 ± 4.92	21.98 ± 6.92	<0.001
a	0.66 ± 4.03	1.01 ± 4.34	2.28 ± 4.04	0.091
b	9.54 ± 5.80	9.53 ± 4.67	4.49 ± 6.92	<0.001
Red	100.94 ± 14.33	78.38 ± 9.98	58.41 ± 12.34	<0.001
Green	93.38 ± 12.69	71.16 ± 13.14	51.60 ± 16.13	<0.001
Blue	78.92 ± 15.15	57.53 ± 14.52	46.33 ± 17.81	<0.001
RGB-SUM	273.23 ± 36.99	207.08 ± 32.36	156.34 ± 43.06	<0.001
HSV	0.40 ± 0.06	0.33 ± 0.06	0.24 ± 0.05	<0.001
HSL	35.47 ± 4.85	26.82 ± 4.08	20.44 ± 5.57	<0.001

Significant difference at *p* ≤ 0.001; ^i^ farms with a low prevalence of SARA; ^ii^ farms with a moderate prevalence of SARA; ^iii^ farms with a high prevalence of SARA; L: color lightness unit ranged from 0 to 100. a: redness/greenness of the color unit ranged from −128 to 127. b: yellowness/blueness of the color unit ranged from −128 to 127. Red, Green, and Blue unit ranged from 0 to 255 in an 8-bit per channel representation. RGB-SUM is labeled as the summary of the Red, Green, and Blue color components; HSV: hue, saturation, and value. Value unit is a percentage (0% to 100%). HSL: hue, saturation, and lightness. Lightness unit is a percentage (0% to 100%).

**Table 4:** Comparison of histological measurements in the layers of the ruminal epithelium (mean ± SD).

Ruminal Epithelium	Category 1 ^i^ (*n* = 15)	Category 2 ^ii^ (*n* = 15)	Category 3 ^iii^ (*n* = 15)	*p*-Value
Stratum corneum (μm)	33.67 ± 12.83	43.07 ± 24.97	41.67 ± 16.25	0.342
Connective tissue (μm)	103.27 ± 39.35	135.00 ± 61.32	148.13 ± 63.83	0.090
Non-keratinized epithelium (μm)	80.13 ± 14.46	116.47 ± 28.64	166.67 ± 21.41	<0.001
Total epithelium (μm)	217.07 ± 51.69	294.53 ± 61.26	356.47 ± 74.08	<0.001

Significant difference at *p* ≤ 0.001; ^i^ farms with a low prevalence of SARA; ^ii^ farms with a moderate prevalence of SARA; ^iii^ farms with a high prevalence of SARA.

## Data Availability

All data generated for this study are presented within this manuscript.
